# MASCC/ISOO clinical practice statement: Current understanding on controversies in basic oral care in hemato-oncology and hematopoietic cell transplantation

**DOI:** 10.1007/s00520-024-08690-1

**Published:** 2024-07-25

**Authors:** Judith E. Raber-Durlacher, Yehuda Zadik, Nathaniel S. Treister, Noa Stempler, Julia S. Bruno, Joel B. Epstein, Sharon Elad

**Affiliations:** 1 Department of Oral Medicine, Academic Center for Dentistry Amsterdam (ACTA), University of Amsterdam and VU University, Gustav Mahlerlaan 3004, 1081 LA Amsterdam, The Netherlands; 2 Department of Oral and Maxillofacial Surgery, Amsterdam UMC, University of Amsterdam, Amsterdam, The Netherlands; 3 Department of Oral Medicine and Saligman Clinics, Faculty of Dental Medicine, The Hebrew University of Jerusalem, and Hadassah Medical Center, Jerusalem, Israel; 4 Division of Oral Medicine and Dentistry, Brigham and Women’s Hospital, Boston, MA USA; 5 Department of Oral Medicine, Infection and Immunity, Harvard School of Dental Medicine, Boston, MA USA; 6 Oral Medicine Unit, Sheba Medical Center, Tel Hashomer, Israel; 7 Molecular Oncology Center—Hospital Sírio-Libanês, São Paulo, Brazil; 8 Dental Oncology Service, City of Hope Comprehensive Cancer Center, Duarte, CA and Cedars-Sinai Health System, Los Angeles, CA USA; 9 Oral Medicine, Eastman Institute for Oral Health, University of Rochester Medical Center, Rochester, NY USA

**Keywords:** Basic oral care, Hematopoietic cell transplantation, Hematologic malignancies, Oral complications, Controversies

## Abstract

**Purpose:**

A MASCC/ISOO Clinical Practice Statement (CPS) is aimed at generating a concise tool for clinicians, which concentrates on practical information needed for the management of oral complications of cancer patients. This CPS is focused on the current understanding of controversies that may arise while providing basic oral care in hemato-oncology patients and hematopoietic cell transplantation recipients (HCT). The CPS will summarize and elucidate controversies that have appeared in the literature and professional discussions.

**Methods:**

This CPS was developed based on a critical evaluation of the literature followed by a structured discussion of a group of leading experts, members of the Oral Care Study Group of MASCC/ISOO. The information is presented in the form of succinct bullets to generate a short manual about the best standard of care.

**Results:**

Controversies about the use of chlorhexidine (CHX) oral rinse, mechanical dental plaque removal procedures, the need for toothbrush replacement during phases of low blood cell counts, and the use of lidocaine mouthwash for oral pain were identified and discussed. Consensus about the best standard of care was outlined.

**Conclusion:**

The following ratifications are applicable for oral care in hemato-oncology patients and patients undergoing HCT: (1) CHX may reduce the risk of oral infections, although it was not found to reduce the risk of oral mucositis. (2) Toothbrushing and proficient interproximal cleaning should not be discouraged during HCT. (3) Toothbrushes do not need to be replaced daily and are preferred over cleansing swabs. (4) Lidocaine rinse, swish and spit, may be considered to palliate oral mucosal pain if applied in a certain manner.

**Supplementary Information:**

The online version contains supplementary material available at 10.1007/s00520-024-08690-1.

## Introduction

Hemato-oncology patients and hematopoietic cell transplantation (HCT) recipients often develop oral complications [1, 2]. These can manifest in the oral mucosa, salivary glands, the periodontium and the dentition, and musculoskeletal tissues. Complications such as bleeding and infection may manifest at multiple oral sites. As these acute and long-term oral sequelae of treatment with chemotherapy and HCT may cause significant morbidity and in some cases mortality, basic oral care in these patients is vital.

Basic oral care comprises the activities that should be part of the patient’s routine care before, during, and after cancer treatment in order to maintain good oral health. A position paper on basic oral care for hemato-oncology patients and HCT recipients has been published by the joint task force of the Multinational Association of Supportive Care in Cancer/International Society of Oral Oncology (MASCC/ISOO) and the European Society for Blood and Marrow Transplantation (EBMT) [2]. The protocol proposed in this joint publication addresses the following fundaments of basic oral care: infection prevention and control of systemic spread, pain control, maintaining oral function, and maintaining quality of life. The dental professional procedures that need to be performed before hematological treatments are detailed in a separate CPS dedicated to this topic.

Over the years, a number of controversies have appeared in the literature and in professional discussions related to some basic oral care interventions for hemato-oncology patients and HCT recipients. Therefore, the MASCC/ISOO Oral Care Study Group (OCSG) composed a multi-professional working group to summarize and elucidate these controversies.

## Objective

The present Clinical Practice Statement (CPS) of the MASCC/ISOO OCSG will outline controversies that may arise while providing basic oral care in hemato-oncology patients and HCT recipients and will summarize the best standard of care.

## Methods

Common controversies were listed, and pertinent literature was reviewed and discussed in a multi-step process by an international and multi-professional working group of the OCSG of MASCC/ISOO. During the development of the manuscript, point questions that deemed a closer look were generated, and a literature search was done to ensure the accuracy of the information. The draft CPS was reviewed and approved by two independent boards: The ISOO Advisory Board and the MASCC Guidelines Committee. The Statement follows the MASCC/ISOO Guidelines Policy.

## Controversy 1: the role of chlorhexidine oral rinse in cancer patients

- There is confusion about the use of chlorhexidine (CHX) oral rinse for basic oral care in cancer patients. This confusion is related to a presumed conflict between the role of CHX in oral care and a MASCC/ISOO clinical practice guideline for the management of oral mucositis.
- CHX mouth rinse is a broad-spectrum antiseptic agent in widespread use for pharmacologic dental plaque control and oral microbial load reduction. It is available in different concentrations and as an either alcohol-free solution or an alcohol-based elixir. CHX is active against Gram-positive and Gram-negative bacteria, yeasts, and some lipophilic viruses [3]. Numerous studies confirmed the efficacy of CHX mouth rinses to reduce plaque accumulation, thereby reducing periodontal inflammation. The best effect of CHX rinse is expected when CHX rinse is used in combination with mechanical plaque removal [4]. Likewise, CHX anti-fungal effect was demonstrated in in vitro and in clinical studies [5, 6]. Furthermore, by reducing oral inoculum, CHX likely reduces the risk of bacteremia [7].
- Common side effects include transient discoloration of the teeth and tongue, taste disturbance, and less commonly oral irritation or hypersensitivity.
- The MASCC/ISOO clinical practice guidelines for the management of oral mucositis recommended against the use of CHX mouth rinse for the prevention of oral mucositis in patients undergoing head and neck radiotherapy [8]. This was based on a systematic review that identified evidence for lack of efficacy to prevent oral mucositis in these patients [9]. No guideline was possible for the use of CHX for the prevention or treatment of oral mucositis in any other population of cancer patients due to insufficient and/or a lack of evidence [10].
- Although the role of CHX in the management of oral mucositis in hemato-oncology patients and HCT recipients is not yet fully elucidated, rinsing with CHX oral solution might be beneficial for plaque control and reducing gingival inflammation in these patients [4].
- Best standard of care:
  - CHX may be considered in cancer patients who are at high risk for oral infections.
  - CHX oral rinse is advised in cancer patients in whom mechanical removal of the oral microbial biofilm by toothbrushing/interproximal cleaning is compromised.
  - By reducing the oral microbial load, CHX may contribute to the prevention of oral and possibly systemic infections during neutropenia.
  - CHX rinse provides the optimal effect when combined with mechanical plaque removal.

## Controversy 2: toothbrushing and interproximal plaque removal during leukopenia or thrombocytopenia

- Some controversy exists about advising patients to continue with toothbrushing and interproximal plaque removal during leukopenia, neutropenia, or thrombocytopenia. This is based on the theoretical concern of inducing bacteremia and gingival bleeding when leukocyte, neutrophil, or thrombocyte counts are low, respectively.
- There is robust evidence in the dental literature that the accumulation of dental plaque induces gingival inflammation associated with bleeding [11].
- Furthermore, in the presence of gingivitis, normal oral functions such as chewing often result in bacteremia [12]. Thus, reducing plaque accumulation aimed to prevent and ameliorate gingival inflammation is paramount to minimize the risk of gingival bleeding, bacteremia, and systemic infectious complications.
- Best standard of care:
  - It is advised for patients to continue toothbrushing during leukopenia, neutropenia, or thrombocytopenia. To minimize the risk of bleeding, the patient may use a toothbrush classified as “soft” or “very soft,” either manual or electric. Practicing this twice daily will reduce plaque deposits and thus reduce the risk of gingival inflammation and bleeding.
  - Patients who are able to use interproximal cleaning devices (e.g., dental floss, tooth picks, or interdental brushes) proficiently should not be discouraged from continuing their use during episodes of low leukocyte and/or thrombocyte counts. Interdental cleaning should be carried out carefully, so as not to injure the tissue, preferably using waxed floss or flexible tooth picks.
  - However, it should be noted that for inexperienced patients who do not routinely use interproximal cleaning devices, episodes of leukopenia and/or thrombocytopenia are not the best times to initiate their use.
  - Patients should be educated on the importance of maintaining good oral hygiene throughout their cancer treatment and receive instructions on efficient and atraumatic oral hygiene procedures.
  - Painful oral mucositis may limit toothbrushing or interproximal cleaning of the dentition. In such cases, topical anesthetics/analgesics to control mucositis-associated pain may facilitate these procedures, and use of alcohol-free CHX rinse may be used as an adjunct to support oral care.

## Controversy 3: toothbrushes should be replaced by oral care swabs during leukopenia/thrombocytopenia

- Some controversy exists whether toothbrushes should be replaced by oral care swabs during leukopenia/thrombocytopenia in order to decrease the risk of inducing bacteremia and/or gingival bleeding.
- Oral care cleansing swabs are disposable, single-use oral care sponges or soft foam attached to a stick, also known as sponge swabs or foam swabs. These applicators are used to remove gross debris and improve moistening [13]. They are not intended to substitute toothbrushing for effective plaque control. They are often in use in hospital settings for patients who are unable to perform oral hygiene.
- Oral care foam-type swabs have been shown to be ineffective for the removal of dental plaque and the prevention of gingival inflammation. Soaking the foam-type swab in 0.2% CHX solution increases its efficacy in plaque control and reducing gingivitis [13–15].
- Best standard of care:
  - Toothbrushes are preferred over oral care cleansing swabs for removing dental plaque. A soft or ultra-soft toothbrush is recommended.
  - Oral care cleansing swabs can be considered when there is a need to moisten the oral tissues and clear the oral surface of gross debris and thickened saliva as an adjunct to toothbrushing and interdental cleaning.
  - In exceptional cases in which a patient is unable to use or tolerate a toothbrush, caregivers may consider using an oral care swab soaked in CHX solution for a limited period until a toothbrush can be used. In intubated patients, oral care swabs may be used to perform oral hygiene.

## Controversy 4: toothbrushes should be replaced daily during leukopenia

- Some controversy exists whether toothbrushes should be replaced daily when leukocyte or neutrophil counts are low.
- Toothbrushes become contaminated with bacteria as soon as they are used. It has been proposed that toothbrushes may be a source of infection, particularly in neutropenic patients. For this reason, it has been suggested that there is a benefit for replacing toothbrushes daily.
- Best standard of care:
  - A toothbrush does not need to be replaced daily. In general, the toothbrush should be replaced when the first signs of wearing of the bristles become visible, usually after 2–3 months.
  - After toothbrushing, the toothbrush should be rinsed thoroughly with tap water and allowed to air dry and, thus, not stored in a container.
  - When interdental brushes are used, these should be thoroughly rinsed with tap water as well and allowed to air dry.

## Controversy 5: lidocaine mouthwash for management of oral pain

- There is a discussion of whether lidocaine hydrochloride anesthetic mouthwash should be advised in patients suffering from mucositis-associated oral pain.
- Lidocaine viscous solution is commonly used as a single agent or as a part of a compound for relieving oral pain. Overall, it is tolerated very well, although a minority of patients may report a transient burning sensation when using it.
- The manufacturer information sheet includes an instruction against using lidocaine rinse within 60 min prior to a meal. The concern is that the lidocaine rinse may increase the risk of aspiration [16].
- However, when used properly, lidocaine solution may facilitate food intake and oral function in patients with painful oral mucositis. In practice, lidocaine is commonly used in the setting of oral pain in cancer patients.
- Best standard of care:
  - Lidocaine rinse, swish and spit, may be considered to palliate oral mucosal pain in cancer patients. It is advised to inform the patient about the expected numbness sensation prior to the first rinse. Additionally, it is advised to instruct the patient to avoid gargling with the solution in order to avoid possible sensation of loss of control of swallowing.
  - In cases of solitary painful ulcers, local application with a cotton swab may be considered instead of a rinse.
  - To the best of our knowledge, there are no Pubmed citations regarding aspiration or choking due to using lidocaine mouth rinse. Furthermore, the effect of lidocaine on oral pain relief is of short duration and will not last until 60 min after the rinse. Therefore, if the oral pain necessitates opioids or other systemic pain medications, or oral intake is compromised, or insertion of a feeding tube is the alternative to lidocaine rinse, it is at the clinician’s and patient’s discretion whether to use the lidocaine mouth rinse prior to the meal.
  - Systemic absorption in patients with oral ulceration has been reported; however, these levels are far below the anti-arrhythmic therapeutic window [17]. Despite systemic absorption being minimal, it is advised to limit the total number of daily lidocaine rinses to the lowest dosage that results in effective anesthesia, especially in children.
  - Some patients may develop adverse effects of lidocaine mouthwash. The list of potential adverse effects can be found in the manufacturer brochure or online resources for professionals.
  - Rinsing with lidocaine solution is not advised in children under 3 years old.

### Supplementary Information

Below is the link to the electronic supplementary material.

Supplementary file1 (DOCX 23.7 KB)

## Data Availability

No datasets were generated or analyzed during the current study.

## Declarations

### Disclaimer

The MASCC/ISOO OCSG Statements are developed to facilitate the expert-opinion based management of oral complications where high-quality evidence is lacking. Clinicians should use their judgment in making treatment decisions for individual patients. The Statement authors and the MASCC/ISOO do not guarantee or take responsibility for clinical outcomes in individual patients.
