# Supplementary material for: MASCC/ISOO clinical practice statement: Current understanding on controversies in basic oral care in hemato-oncology and hematopoietic cell transplantation
Source: Support Care Cancer. 2024 Jul 25;32(8):550. doi: 10.1007/s00520-024-08690-1 (PMC11269443; doi:10.1007/s00520-024-08690-1)
Supplement: Supplementary file 1 — Supplementary file1 (DOCX 23.7 KB) [file 520_2024_8690_MOESM1_ESM.docx]

MASCC/ISOO Clinical Practice Statement: Current understanding on controversies in basic oral care in hemato‑oncology and hematopoietic cell transplantation

**Suggested Readings:**

1. Cardona A, Balouch A, Abdul MM, Sedghizadeh PP, Enciso R. Efficacy of chlorhexidine for the prevention and treatment of oral mucositis in cancer patients: a systematic review with meta-analyses. J Oral Pathol Med. 2017 Oct;46(9):680-688.
2. Poppolo Deus F, Ouanounou A. Chlorhexidine in Dentistry: Pharmacology, Uses, and Adverse Effects. Int Dent J. 2022 Jun;72(3):269-277.
3. Potting CMJ, van Leeuwen SJM, Kurstjens MH, Bronkhorst EM, Thomas RZ, Blijlevens NMA, Huysmans MDNJM. A randomized controlled trial of manual versus powered tooth brushing during haematopoietic stem cell transplantation. Oral Dis. 2022 Oct;28(7):1987-1994.
4. Pearson LS, Hutton JL. A controlled trial to compare the ability of foam swabs and toothbrushes to remove dental plaque. J Adv Nurs. 2002 Sep;39(5):480-9. 10.1046/j.1365-2648.2002.02313.x. PMID: 12175357.
5. https://www.cdc.gov/oralhealth/infectioncontrol/faqs/toothbrush-handling.html#:~:text=In%20removing%20plaque%20and%20other,with%20tap%20water%20following%20brushing. Accessed 15 April 2024
6. Yamashita S, Sato S, Kakiuchi Y, Miyabe M, Yamaguchi H. Lidocaine toxicity during frequent viscous lidocaine use for painful tongue ulcer. J Pain Symptom Manage. 2002 Nov;24(5):543-545.
